# Roles of Small-Molecule Compounds in Plant Adventitious Root Development

**DOI:** 10.3390/biom9090420

**Published:** 2019-08-28

**Authors:** Yuzheng Deng, Chunlei Wang, Ni Wang, Lijuan Wei, Weifang Li, Yandong Yao, Weibiao Liao

**Affiliations:** College of Horticulture, Gansu Agricultural University, Lanzhou 730070, China

**Keywords:** small signaling molecules, interactions, gene expression

## Abstract

Adventitious root (AR) is a kind of later root, which derives from stems and leaf petioles of plants. Many different kinds of small signaling molecules can transmit information between cells of multicellular organisms. It has been found that small molecules can be involved in many growth and development processes of plants, including stomatal movement, flowering, fruit ripening and developing, and AR formation. Therefore, this review focuses on discussing the functions and mechanisms of small signaling molecules in the adventitious rooting process. These compounds, such as nitric oxide (NO), hydrogen gas (H_2_), hydrogen sulfide (H_2_S), carbon monoxide (CO), methane (CH_4_), ethylene (ETH), and hydrogen peroxide (H_2_O_2_), can be involved in the induction of AR formation or development. This review also sums the crosstalk between these compounds. Besides, those signaling molecules can regulate the expressions of some genes during AR development, including cell division genes, auxin-related genes, and adventitious rooting-related genes. We conclude that these small-molecule compounds enhance adventitious rooting by regulating antioxidant, water balance, and photosynthetic systems as well as affecting transportation and distribution of auxin, and these compounds further conduct positive effects on horticultural plants under environmental stresses. Hence, the effect of these molecules in plant AR formation and development is definitely a hot issue to explore in the horticultural study now and in the future.

## 1. Introduction

The root system of a plant is made up of the primary, lateral, and adventitious roots. The primary root originates during embryogenesis and will later elongate after germination. The AR is initiated and developed post-embryonically from differentiated cells; however, the later root is derived from pericycle cells, which is also defined anatomically as meristems [1,2]. AR is a type of lateral root, which is originally derived from stems and leaf petiole [3]. The process of AR formation can be divided into three phases: induction (when biochemical and molecular changes occur), initiation (when cells start to divide to form an internal root meristem), and expression (when the AR primordium grows and emerges from the stem) [4]. The development of AR is a vital step in the vegetative propagation of plants, which is regulated by various endogenous factors such as plant hormones, metabolic constituents, enzyme activities, and external environmental stimulations, and it plays a key function for plant adaptation to abiotic stresses, including salt, drought, heavy metal, and osmotic stresses [5]. Moreover, AR would be subjected to biotic stress, such as beneficial or pathogenic microbes during plant growth, and in AR development of artificial explants produced by wounding [6]. Therefore, understanding the mechanism of adventitious rooting is of significant importance to strategize breeding efforts to maximize its marketable yield [7]. 

Many different kinds of molecules transmit information between the cells of multicellular organisms, including small-molecule compounds. These molecules are produced by signaling cells and subsequently bind to receptors in target cells, acting as ligands and chemical signals to coordinate responses and a variety of biological processes both in animals and plants [8,9]. Over the past decades, carbon monoxide (CO) and hydrogen sulfide (H_2_S) were regarded as toxic molecule compounds in animals. Gradually, small signaling molecules have proven to be resistant to various abiotic stresses in plants, such as chilling, osmotic stress, drought stress, flooding, salt stress, heavy metal stress (aluminum stress, cadmium toxicity, and mercury toxicity), and UV-A irradiation [5,7,10]. NO was also discovered to influence the interactions between plants and microbes during pathogenesis [11,12]. Besides, they are involved in numerous plant growth and development processes, including cell division, stomatal movement, adventitious rooting, flowering, fruit ripening and development, and seed dormancy or germination [5,13,14,15]. In recent years, increasing kinds of small molecules have been found to participate in signal transduction during adventitious rooting. For example, some typical small molecules such as NO, H_2_, CH_4_, ethylene (ETH), and H_2_O_2_ have been indicated to induce AR formation and development in plants [16,17,18,19]. Also, the roles of small compounds under stresses have been widely investigated through studying their functions and the crosstalk among them. Now, multiple researchers suggest that small-molecule compounds can promote AR formation and development via mediating a variety of growth and developmental processes, which help to improve the resistance against environmental stress conditions in plants. 

AR development is a complicated process and influenced by many abiotic factors such as mineral nutrition (Ca, N, Zn), light, temperature, and various biotic factors including ectomycorrhizas and agrobacterium rhizogenes [1,20]. In addition, previous reviews and studies have reported that multiple endogenous factors (aging, polyamines, enzymatic activities of peroxidases, phytohormones, and phenolic compounds) can affect the development of AR [1,20]. Furthermore, the innovational application of small-molecule compounds is found to be necessary for promoting AR development. A number of studies have demonstrated the effectiveness of these compounds when applied to multiple horticultural plant species such as cucumber, marigold, and tomato. However, there is little review about the effects of small-molecule compounds in plant AR development. The innovational application of small-molecule compounds is necessary to promote AR formation, and its great importance in horticultural study cannot be ignored. For a more in-depth study of the effects of ARs on small molecules, this review will mainly discuss the recent progresses of small-molecule compounds on AR formation and development in plants. Also, the interactions between NO and other signaling molecules are also discussed.

## 2. Functions of Small-Molecule Compounds during Adventitious Rooting 

### 2.1. Nitric Oxide (NO) 

NO plays a crucial role as a second messenger molecule, after Ca^2+^, of plant signal transduction in the growth and development of plants, such as seed germination and dormancy, stomatal movement, and photosynthesis [21]. NO also participates in plant responses to various stresses, including heavy metal, low temperature, drought, salt, and UV-B radiation [22]. 

In plants, NO is synthesized through two synthetic pathways: enzymatic and nonenzymatic. The enzymatic pathway includes nitrate reductase (NR) and NO synthase (NOS)-like enzymes. Previous studies of NO showed that NR played crucial roles in the process of physiological activities in NO production [10]. It was also reported that both NR and NOS could contribute to NO production and synergistically induced adventitious rooting in marigold (*Tagetes erecta* L. ‘Marvel’) [23] and AR development in cucumber (*Cucumis sativus* L.) [10], suggesting that NO could be produced by NOS substrates in plants and that NOS-like activity does exist in plants. The treatment of 10 μM NO donor sodium nitroprusside (SNP) significantly promoted AR formation in cucumber explants through NOS and NR pathways [24,25]. Additionally, cyclic guanosine monophosphate (cGMP) was involved in NO-induced adventitious rooting of marigold explants to serve as a crucial component of the NO-regulated signaling pathway [23]. Ample evidence also showed that exogenous NO dramatically triggered adventitious rooting in marigold [26,27]. These findings indicate that NOS and NR are important signaling molecules in the production of NO to promote adventitious rooting.

It has been widely reported that NO may regulate the developmental process of adventitious rooting. Application of NO significantly enhanced AR length and number in the cuttings of ground-cover chrysanthemum (*Dendranthema morifolium* ‘Beiguozhicun’), and the explants of cucumber and marigold [26,27,28], which indicates that NO has a positive effect on AR development in plants.

In addition, cell cycle regulation plays important roles in the xylem during the growth of plant roots. Cell cycle regulation in the xylem pericycle plays an important part in root organogenesis. Cell cycle regulation often occurs in the xylem pole of the pericycle, in which cells proceed to the G2 phase, whereas the rest of cells in pericycle remain at the G1 phase, which indicates that NO is involved in cell cycle regulation in the process of adventitious rooting in plants [7]. 

NO can also regulate AR development through adjusting enzyme activities, such as peroxidase (POD), polyphenol oxidase (PPO), and indoleacetic acid oxidase (IAAO). Furthermore, NO accumulation significantly increased the activity of pro-oxidants including triphosphopyridine nucleotide (NADPH oxidase) and antioxidants such as superoxide dismutase (SOD), catalase (CAT), POD, ascorbate peroxidase (APX), dehydroascorbate reductase (DHAR), and glutathione reductase (GR) enzymes in AR development in mountain ginseng (*Panax ginseng* C.A. Meyer) [29]. NO donor SNP increased PPO activity and reduced POD and IAAO activities during the AR development process in cucumber [30]. Exogenous NO could also increase IAAO, POD, and PPO activities in marigold, which was associated with the induction of AR [27,28]. These results indicate that NO triggers AR development by regulating enzymatic activities.

NO also regulates AR development under abiotic stresses. Exogenous NO enhanced the root number and root fresh weight in marigold explants under drought stress [16]. And NO donor SNP prompted the adapted mesophyll cell ultrastructure changes under drought conditions. Meanwhile, application of NO remarkably increased the chlorophyll content and fluorescence energy parameters, including Fv/Fm, ɸPSII, and qP parameters, and inhibited the decrease of water-soluble carbohydrate (WSC) and total soluble protein, which subsequently promoted rooting under drought stress [16]. Recently, the NO donor was reported to significantly induce AR length and number in cucumber explants under osmotic stress by strengthening the photosynthetic performance [22]. And NO treatment increased the ψ_w_, SOD, CAT, and APX activities and chlorophyll content in cucumber explants [22]. Moreover, NO significantly induced new AR formation in rice (*Oryza sativa* L. cv. Komal) seedlings under arsenate (As^V^) stress by increasing APX content [31]. Therefore, NO can trigger AR development and play an important role during the process of AR development under abiotic stresses.

### 2.2. Hydrogen Gas (H_2_) and Hydrogen Sulfide (H_2_S)

Hydrogen gas (H_2_) is a colorless and tasteless molecule, and hydrogen is one of the most abundant elements in the universe, constituting nearly 75% of the mass of the universe [32]. It has been widely reported in animals that H_2_ has come to the forefront in therapeutic medical gas research. Recently, the attention on the role of H_2_ has changed from animals to plants. It has been found that H_2_ could regulate the growth and development of plants, including seed germination, seedling growth, stomatal closure. and root elongation [33]. In addition, H_2_ enhances the tolerance of plants to abiotic stress conditions such as salt stress, osmotic stress, drought, cadmium toxicity, aluminum stress, mercury toxicity, and UV-A irradiation [5].

Many studies have shown that H_2_ has a positive effect on adventitious root development in plants. In cucumber, 50% hydrogen-rich water (HRW) significantly induced adventitious rooting and enhanced NO content in a time-dependent manner, which reached a maximum at 24 h during the treatment. Also, the application of H_2_ triggered transition from G1 to S phase in plant cell cycle to enter a new cell cycle in a synchronous manner [7]. And HRW remarkably induced the activities of antioxidant enzymes, such as POD, PPO, and IAAO [30]. Further studies reported that 50% HRW could prompt AR development in marigold explants by increasing relative water content (RWC) and WSC, starch, and soluble protein content, as well as POD, PPO, and IAAO activities, and it could also decrease stomatal aperture and electrolyte leakage [5]. 

Also, H_2_ exerted a positive effect on AR development under stress conditions. Fifty percent HRW significantly induced AR length and number and enhanced the RWC during rooting in cucumber explants under drought stress [18]. Also, exogenous H_2_ dramatically increased leaf chlorophyll content, Fv/Fm, ɸPSII, and qP as well as the activities of SOD, POD, CAT, and APX enzymes during drought [18]. Additionally, a recent study showed that under cadmium (Cd) stress, 50% HRW was the most proper dose to trigger adventitious rooting in cucumber [34]. Compared with Cd treatment, HRW + Cd treatment significantly reduced the content of malondialdehyde (MDA), hydrogen peroxide (H_2_O_2_), superoxide radical (O_2_^−^), thiobarbituric acid reactive substances (TBARS), ascorbic acid (AsA), and reduced glutathione (GSH), as well as relative electrical conductivity (REC), lipoxygenase (LOX) activity, AsA/docosahexaenoic acid (DHA) ratio, and GSH/oxidized glutathione (GSSG) ratio, while increasing DHA and GSSG content. HRW + Cd treatment also significantly increased in the activity and related gene expressions of *APX*, *DHAR*, *monodehydroascorbate reductase* (*MDHAR*), and *GR*. Additionally, HRW + Cd treatment increased the contents of osmotic adjustment substances, as well as the activities of POD and PPO, while significantly decreasing IAAO activity [34]. These results confirm that H_2_ induces adventitious rooting under Cd stress by decreasing the oxidative damage, increasing osmotic adjustment substance content, and regulating rooting-related enzyme activity.

Hydrogen sulfide (H_2_S) is a naturally occurring, colorless, highly soluble, flammable, and toxic gas [35]. It has been also considered as the third gaseous transmitter after NO and CO in animals [36]. Furthermore, exogenous H_2_S has been found to be involved in abiotic stresses of plants such as salinity, drought, extreme temperatures, and heavy metals [37]. Recent research suggested that H_2_S regulated many aspects of plant development, like seed germination and adventitious root induction [38]. The donor of H_2_S, 10 μM sodium hydrosulfide (NaHS) significantly induced AR primordia in cucumber explants [39,40], and 0.2 μM NaHS was suitable for the increase of both root number and root length in excised willow (*Salix matsudana* var. *tortuosa* Vilm) and soybean (*Glycine max* L.) seedlings [38].

### 2.3. Carbon Monoxide (CO) 

CO is a low molecular weight diatomic and poisonous gas that occurs ubiquitously in nature. However, CO has recently been proven to be one of the most essential cellular components and regulates a variety of biological processes both in animals and plants. In animals, CO regulates many physiological events such as platelet aggregation, neurotransmission, and vasodilation [18]. Heme oxygenase (HO) is the rate-limiting enzyme in the process of heme catabolism. CO in the human body is mainly produced by the metabolism of HO. There are three types of HO: oxidative stress-inducible heme oxygenase-1 (HO-1), constitutive heme oxygenase-2 (HO-2), and undetermined heme oxygenase-3 (HO-3) [18]. In plants, CO plays a key role in seed germination, stomatal closure, and root development [18], and it is required for the alleviation of abiotic stresses, including salt stress, drought, heavy metal stress, and UV-B radiation [9].

A number of studies showed that CO could trigger AR development. CO treatment induced adventitious rooting in the hypocotyl of mung bean (*Phaseolus radiatus* L. cv. Mingguang) in dose- and time-dependent manners [41]. Also, NO fluorescence was significantly enhanced by the treatment of CO, indicating that CO triggered AR development of hypocotyl cuttings from mung bean seedlings, possibly through regulating the NO/NOS pathway. The CO artificial donor, 10 μM hemin and hematin could significantly induce AR development in cucumber [17,37,42]. Hemin (500 μM) and 30% CO aqueous solution significantly increased the AR number and length in cucumber explants under drought stress [18]. The applied polyethylene glycol (PEG) reduced leaf RWC during adventitious rooting, and CO alleviated the reduction. Furthermore, CO enhanced leaf chlorophyll content and then increased photosynthesis and promoted AR development during drought conditions. Moreover, CO treatment enhanced the activities of SOD, POD, CAT, and APX in cucumber under drought stress [18]. These findings suggest that CO can induce adventitious rooting in cucumber seedlings under drought stress by alleviating the negative effects of drought stress on RWC, chlorophyll content, and antioxidant systems. 

### 2.4. Methane (CH_4_)

CH_4_ is a physiologic inert gas and regarded as an important greenhouse gas in the atmosphere. Recently, studies have illustrated that CH_4_ exhibits anti-apoptotic, anti-inflammatory, and antioxidative activities in animals [43]. CH_4_ also plays an important role in responses of plants to environmental stresses [44]. It was found that aerobic CH_4_ release could be stimulated by the increase of temperature, cutting injuries, production of reactive oxygen species (ROS), and ultraviolet radiation [45,46]. However, only few studies reported the functions of exogenously applied CH_4_ on various plant developmental processes. Eighty percent methane-rich water (MRW) dramatically triggered the increase of AR length and number in cucumber [19]. CH_4_-induced adventitious rooting in cucumber explants required γ-glutamyl cysteine synthetase (γ-ECS) [40,47]. These findings indicate that CH_4_ can be regarded as a crucial inducer during AR process.

### 2.5. Ethylene (ETH)

ETH is a gaseous plant hormone and involved in many growth and development processes in plants, such as seed germination and dormancy, cell division and expansion, leaf senescence, and fruit ripening [48,49]. Zimmerman and Hitchcock (1933) [50] first observed that ETH significantly enhanced the root length and number in AR development. Now, many studies have shown that ETH is involved in AR growth in many plant species [51,52]. In addition, exogenous ETH dramatically increased the activities of antioxidant enzymes (IAAO, POD, and PPO), resulting in AR development in marigold [27]. The ethylene donor ethephon significantly promoted AR development in cucumber and marigold explants [10,27]. But the ethylene precursor 1-aminocyclopropane-1-carboxylic acid (ACC) could not trigger AR formation in wild-type seedlings of *Arabidopsis* (*Arabidopsis thaliana)* [53]. However, ACC was shown to have the effect on adventitious rooting in *Arabidopsis* mutant (*max2* and *max4*) plants in a dose-dependent manner [53]. The application of ACC at 0.01 μM significantly increased the development of AR, but 100 μM ACC exhibited inhibition effects [53]. 

### 2.6. Hydrogen Peroxide (H_2_O_2_)

H_2_O_2_ is viewed mainly as a type of ROS and a signaling messenger. A number of studies reveal that H_2_O_2_ is involved in many biological processes of plants, such as stomatal closure [54], flowering [55], leaf senescence [56], cellular differentiation and plant morphogenesis [57], lateral root formation, and AR development [58,59]. 

According to Bai et al. (2012) [60], in mung bean seedlings, 3-O-C10-HL could stimulate AR formation, depending on cGMP pathways, by triggering endogenous H_2_O_2_ production, which showed that 3-O-C10-HL is likely to participate in auxin-prompted AR formation by H_2_O_2_-dependent cGMP signaling. Li et al. (2007) [59] found that 20–40 μM H_2_O_2_ could significantly increase the number of AR, while treatment with 10–50 μM H_2_O_2_ significantly increased the fresh weight of AR in cucumber. Moreover, in marigold, the treatment of 200 μM H_2_O_2_ significantly induced root length and root number, but its scavenger CAT alleviated its positive effects [23]. It has also been reported that H_2_O_2_ and the synthetic phytohormone indole-3-butyric acid (IBA) may act synergistically to regulate adventitious rooting dependent on the auxin pathway in marigold explants [26]. Exogenous NPA led to a significant decrease in the endogenous levels of auxin, which, in turn, inhibited the endogenous accumulation of H_2_O_2_, suggesting that H_2_O_2_ may be the downstream signal molecule in the auxin signaling cascade [26]. Also, 200 µM H_2_O_2_ promoted the AR length and number in marigold, and treatment with indole-3-butyric acid (IBA) enhanced the endogenous level of H_2_O_2_ in explants [26], which suggests that the promotion of AR by IBA may occur via enhancing the level of H_2_O_2_. In ground-cover chrysanthemum, 200 μM H_2_O_2_ was the most suitable dosage to promote AR development, at which this dosage increased the activities of PPO, IAAO, and WSC, as well as total nitrogen content, and decreased the total polyphenol content [28]. These results indicate that H_2_O_2_ could enhance AR development via increasing some enzyme activities, and carbohydrate and nitrogen contents, and inhibiting polyphenol production. 

Under drought stress, 600 μM H_2_O_2_ treatment significantly promoted root length and number in marigold, attenuated the destruction of the mesophyll cell ultrastructure, and increased leaf chlorophyll content, chlorophyll fluorescence parameters (Fv/Fm, ɸPS II, and qP), and hypocotyl soluble carbohydrate and protein content, while decreasing starch content of marigold [16]. It implies that under drought conditions, the protection of H_2_O_2_ on the ultra-microstructure of mesophyll cells improves the photosynthetic performance of blades and lightens the adverse impacts of drought on the build-up of carbohydrates and nitrogen in explants, boosting adventitious rooting. An overview of the effects and mechanisms in plant AR-related studies of small-molecule compounds under no stress and stress conditions are shown in Table 1 and Table 2, respectively.

## 3. Cross-Talk between Small-Molecule Compounds during Adventitious Root Development

### 3.1. NO and Other Signaling Molecules

#### 3.1.1. NO and CO

NO and CO are two toxic gases, but they function as second messenger molecules in decreasing vascular tone and inhibiting platelet aggregation by simultaneously inducing cGMP content in animals [61]. The biosynthesis enzymes of NO and CO are NOS and HO, respectively, and they share similar isoforms, requirements for activity, regulation, and localization, illustrating the coordinated function of NO and CO in animals [61]. NO and CO also work together to exert growth and development processes in plants, and here we summarize the crosstalk between them during plant adventitious rooting.

In plants, NO and CO are able to act as two typical downstream signals in auxin-induced adventitious rooting. In mung bean seedlings, addition of the CO donor hematin induced AR formation in dose- and time-dependent manners, similarly with the phenotype induced by NO donor SNP. Furthermore, the NO-specific scavenger cPTIO suppressed the entire NO induced by CO, and the root numbers of the mung bean hypocotyls were decreased by NOS inhibitor L-NAME and hematin treatments when compared to that under hematin treatment alone. However, the inhibitor of HO-1 zinc protoporphyrin IX (ZnPPIX) could not affect the SNP action, suggesting that the CO-induced adventitious rooting process is mediated by the NO/NOS pathway, and NO may act downstream of CO during AR formation in mung bean hypocotyls [41].

In cucumber, the *heme oxygenase1* (*CsHO1*) gene was cloned and proved to exhibit HO activity, which was the inducible isoform of heme oxygenase, and it participated in the heme degradation pathway while concomitantly releasing CO. The nitric oxide donor SNP, as well as other AR inducers, could increase the level of the *CsHO1* transcript and the corresponding protein, which further suggested the crosstalk between endogenous HO-1/CO and NO during adventitious rooting and their signal transduction pathway [62]. 

Subsequent research found that application of CO and hemin to cucumber explants induced the production of NO and the NOS-like enzyme inhibitors, and the NO scavenger blocked NO content and AR formation stimulated by CO, but not the NR inhibitors. Simultaneously, researchers found that NO might act as a downstream regulator of CO during adventitious rooting in a cGMP-dependent manner [17].

#### 3.1.2. NO and H_2_

It was reported that NO had a close relation with H_2_ in AR development in cucumber and marigold. The NO scavenger cPTIO, NOS inhibitor NG-nitro-L-Arg methyl ester hydrochloride (l-NAME) or NR inhibitor sodium azide (NaN_3_), and tungstate could partly reduce the effect of H_2_-triggered AR in cucumber, which indicated that NO acts as the downstream signaling molecule, and that NOS and NR might be responsible for NO generation during H_2_-induced AR formation [7]. Moreover, NO and H_2_ induced AR development through regulating the cell cycle and the activity of root organogenesis-related enzymes POD, IAAO, and PPO [30].

#### 3.1.3. NO and ETH

The marigold and cucumber explants treated with ETH and NO together exhibited a better phenotype of AR formation than those treated with ETH or NO alone. These effects of ETH were restrained by cPTIO, L-NAME and NaN_3_, suggesting that the positive role of ETH on AR formation was partially NO-dependent [27]. It was further shown that addition of the ETH donor ethephon increased the endogenous NO level and up-regulated activities of NOS and NR and their corresponding gene expressions in explants, suggesting that the positive role of ETH on AR formation may rely on internal accumulation of NO [10,27]. In marigold, ETH and NO could simultaneously induce the activities of IAAO, POD, and PPO during adventitious rooting [27]. In cucumber, ETH and NO could stimulate AR formation through regulating cell cycle activation, nutrient distribution, and photosynthesis [10]. Together, NO acts as downstream regulator of ETH in AR development.

#### 3.1.4. NO and CH_4_

The applied CH_4_ in cucumber (*C. sativus* ‘Lufeng’) explants triggered generation of NO and was followed by adventitious rooting, which was similar to the effects of the NO donor SNP and NONOate, suggesting the involvement of NO in CH_4_-elicited root organogenesis. Further studies illustrated that CH_4_ induced endogenous NO production through NOS-like enzymes and diamine oxidases (DAOs), and it triggered AR development via controlling expressions of NO-targeted genes [19]. 

#### 3.1.5. NO and H_2_S

In sweet potato (*Ipomoea batatas* L.), application of the H_2_S donor NaHS induced ARs in a dose-dependent manner. Treatment of NaHS increased endogenous H_2_S, IAA, and NO contents, and H_2_S mediated AR, which was blocked by NPA and cPTIO, This indicated that IAA and NO participate in H_2_S-induced adventitious rooting, and this regulation mechanism could also be applicable to root organogenesis in willow (*S. matsudana* var. *tortuosa* Vilm) and soybean (*G. max* L.) stem cuttings [38]

#### 3.1.6. NO and H_2_O_2_

In marigold, previous studies showed that AR length and number were much higher with the application of SNP + H_2_O_2_, compared to SNP or H_2_O_2_ treatment alone. cPTIO, the scavenger of NO, inhibited H_2_O_2_-triggered adventitious root formation, while the treatment of CAT, an H_2_O_2_ inhibitor, suppressed SNP-induced AR development, suggesting that both NO and H_2_O_2_ can have independent or synergistic effects on the induction of adventitious rooting [23]. Further studies indicated that NO and H_2_O_2_ may be downstream signal molecules in the auxin signaling cascade, and NO may be involved as an upstream signaling molecule for H_2_O_2_ production [26].

In ground-cover chrysanthemum, both NO and H_2_O_2_ treatments at the proper dosage increased PPO, IAAO, WSC, and total nitrogen, and they decreased total polyphenol content, which implies that NO and H_2_O_2_ treatments enhanced AR development synergistically and independently by stimulating PPO and IAAO enzyme activities and carbohydrate and nitrogen contents while simultaneously repressing the production of polyphenol [28].

Under drought conditions, SNP and H_2_O_2_ reduced the harmful effects by changing the chlorophyll content, fluorescence parameters, and carbon and nitrogen levels, indicating that H_2_O_2_ and NO act synergistically, and H_2_O_2_ is involved in rooting promoted by NO under drought conditions [16]. 

### 3.2. CO and Other Signaling Molecules

#### 3.2.1. CO and H_2_

In alfalfa seedlings, H_2_ alleviated oxidative stress induced by paraquat via HO-1 signaling, indicating a synergistic effect of H_2_ and CO during plant responses to abiotic stresses [63]. In cucumber, the inducible effect on AR by HRW mimicked that by hemin or HO-1, and the HRW-induced response was restrained by addition of NPA. The blocking of HO-1 inhibitor ZnPPIX and the reversion of CO to HRW-treated AR further suggested that HRW took part in AR formation at least partially through the HO-1/CO directed pathway [64]. In cucumber (*C. Sativus* ‘Xinchun No. 4′), researchers further discovered that CO may be involved in H_2_-triggered adventitious root formation through adjusting RWC, photosynthesis, and metabolic constituent content and alleviating oxidative damage under drought stress conditions [58].

#### 3.2.2. CO and H_2_S

In cucumber, the H_2_S donor NaHS induced adventitious rooting, and the expression of *CsHO-1* as well, and ZnPPIX significantly suppressed the effects induced by NaHS. Furthermore, NaHS regulated similar target genes of HO-1/CO in AR formation, but the H_2_S scavenger hypotaurine (HT) could not influence HO-1/CO AR formation, which suggested that HO-1/CO acts downstream of H_2_S during AR formation [40].

#### 3.2.3. CO and CH_4_

In cucumber, MRW significantly induced AR formation in IAA-depleted explants, and the inducible affect was still effective in soybean and mung bean explants. Meanwhile, auxin signaling-related genes and cell cycle regulatory genes exerted by MRW during AR formation were blocked by ZnPPIX and further reversed by CO, and the involvement of the Ca^2+^ pathway in the MRW-induced AR process was certified. Together, CH_4_ could stimulate adventitious rooting through partially adjusting HO-1/CO and Ca^2+^ pathways [65].

### 3.3. Crosstalk between Other Signaling Molecules

Previous results showed that H_2_S and CH_4_ mediated adventitious rooting in cucumber explants. Further study demonstrated that CH_4_ induced endogenous generation of H_2_S during adventitious rooting, and the H_2_S scavenger HT restrained the development of adventitious root primordia and gene expressions as well as *S*-sulfhydration level elicited by CH_4_, indicating the important role of endogenous H_2_S in participating in CH_4_-induced cucumber adventitious root development [40].

During the adventitious root bioreactor culture of ginseng, the enrichment of O_2_ increased adventitious root mass, ginsenoside contents, and polysaccharides, while CO_2_ and C_2_H_4_ were found to operate opposite impacts as O_2_ on secondary metabolism in plant cell cultures [66]. 

**Figure 1:**
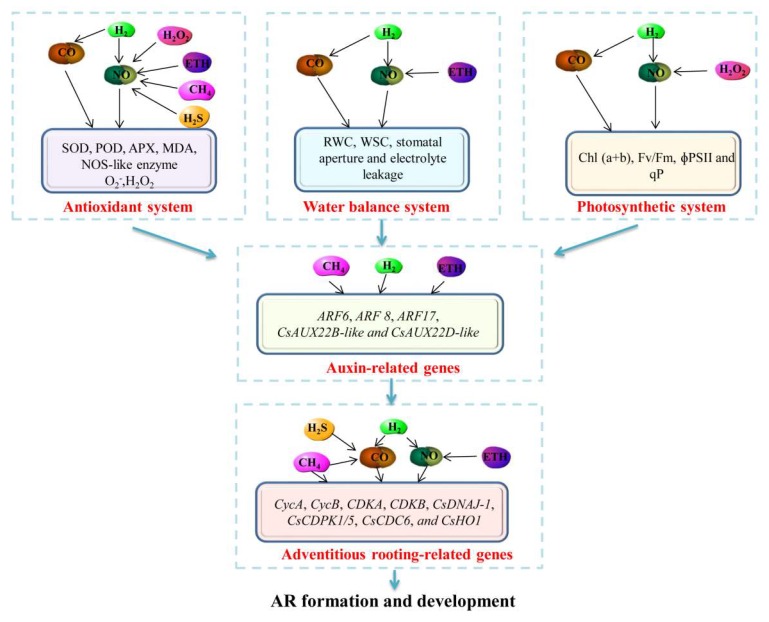
Schematic model of the interactions among different small signaling molecules during plant adventitious rooting through regulating different plant physiological process systems (antioxidant system, water balance system, and photosynthetic system). In the antioxidant system, NO is regarded as the downstream molecule to enhance the enzyme activities of SOD, POD, APX, and MDA and the production of O_2_^−^ and H_2_O_2_ [18,22,23,29,34]. In the water balance system, NO is also located downstream to increase RWC, WSC, stomatal aperture, and electrolyte leakage of plants [5,18,28]. In the photosynthetic system, NO is involved in H_2_, H_2_O_2_, and CO-increased chl (a+b), Fv/Fm, ɸPSII, and qP [16,18,22]. In addition, some auxin-related genes, including *ARF6*, *ARF8*, *ARF17, CsAUX22B-*like, and *CsAUX22D-*like, can be up-regulated by the application of CH_4_, H_2_, and ETH [40,47,64,67]. Furthermore, small signaling molecules up-regulate AR-related genes such as *CycA*, *CycB*, *CDKA*, *CDKB*, *CsDNAJ-1*, *CsCDPK1/5*, *CsCDC6,* and *CsHO1* [7,10,17,39,40,47,62,64,65] and finally conduct the formation and development of adventitious roots.

In summary, H_2_, NO, CO, ETH, and other small-molecule compounds work together by regulating antioxidant, water balance, and photosynthetic systems, and they affect the transportation and distribution of auxin as well as expedite plant adventitious rooting. The mechanisms of the cross-talk between NO and other small signal molecules during plant adventitious rooting are summarized in Figure 1.

## 4. Some Related Genes during Adventitious Root Development Induced by Small-Molecule Compounds

It is well known that cell cycle regulation genes are often related to the regulation of cell division in plant internode growth. Two *cyclins* (*CycA*, *CycB*) and two *CDK* (*CDKA* and *CDKB*) genes are found to analyze the effect of cell division on AR formation in plants. In cucumber, NO and H_2_ treatment up-regulated the expression of the cell-cycle regulatory genes *CycA*, *CycB*, *CDKA,* and *CDKB*, which participate in mediating cell division in internode growth and root meristem induction to boost the process of AR development. This result suggests that both NO and H_2_ take part in regulating the cell cycle progress to the mitotic phase [7]. Also, NO and ETH treatments enhanced the transcription levels of *CsCDPK1* and *CsCDPK5* genes in cucumber explants [64]. The application of CH_4_ could also up-regulate cell cycle regulatory genes in cucumber, including *CsCDC6*, *CsCDPK1*, *CsCDPK5*, and *CsDNAJ-1* [40,47].

Furthermore, molecular evidence illustrates that the corresponding genes of DNAJ-like proteins and calcium-dependent protein kinases (CDPKs) are related to the initiation and development of adventitious roots. Xu et al. (2017) [10] found that NO and ETH could induce AR development in cucumber by regulating *CsDNAJ-1* and *CsCDPK1/5* genes. However, when NO and ETH were applied alone, the relative expressions of *CsDNAJ-1*, *CsCDPK1,* and *CsCDPK5* genes were significantly higher than that of any other treatments. The *CsDNAJ-1* and *CsCDPK1/5* target genes were up-regulated by the application of H_2_ and HO-1/CO during AR development in cucumber [64]. The H_2_S donor NaHS triggered up-regulation of target genes responsible for HO-1/CO-induced cucumber adventitious root formation, including *CsDNAJ-1* and *CsCDPK1/5* [39]. *CsDNAJ-1* and *CsCDPK1/5* genes were the possible target genes in the MRW-induced HO-1/CO-mediated cucumber adventitious development [66]. Also, the up-regulation of the HO-1 gene *CsHO1* can inhibit antioxidant processes in some abiotic stresses. *CsHO1* was found to be involved in cucumber adventitious rooting via NO, H_2_S, and CO treatments [17,62]. In addition, there are also some adventitious rooting-related genes. For example, the transcripts of *CsmiR160* and *CsmiR167* were increased or decreased, respectively, by the application of MRW, indicating that CH_4_ may play a positive effect on AR development in cucumber [47]. Therefore, NO, ETH, H_2_, CO, H_2_S, and CH_4_ are able to induce high expressions of the *CsDNAJ-1*, *CsCDPK1/5,* and *CsHO1* genes to mediate adventitious rooting.

Besides, some auxin-response genes are associated with AR formation. Previous studies showed that the auxin response factors *ARF6* and *ARF8*, which were targeted by *miR167*, had important effects on the regulation of AR development by adding ETH in *Arabidopsis*; however, *ARF17*, a target of *miR160*, was a negative regulator [67]. In cucumber, *CsAUX22B-*like and *CsAUX22D-*like were target genes to HRW-induced AR formation [64]. Recently, MRW could increase the transcripts levels of *CsAux22D-*like and *CsAux22B-*like in cucumber explants [47]. Also, CH_4_ could induce two auxin-signaling genes, such as *CsAux22D-*like and *CsAux22B-*like, which are responsible for AR formation in cucumber [40]. Table 3 summarizes all genes regulated by small-molecule compounds during adventitious rooting in plants.

## 5. Conclusions and Perspectives

In recent years, there have been increasing studies about the effects on the small signaling molecules of AR formation and development. Some molecules, like CO and H_2_S, were previously considered as toxic. They recently were assumed to be the gaseous substances to induce AR development. This review is based on a number of studies to show that small-molecule compounds such as NO, H_2_, H_2_S, CO, CH_4_, and ETH can be necessary to trigger adventitious rooting. However, the mechanisms of these compounds in adventitious rooting should still be illustrated and consummated. Many results have reported that some small molecules exert interactions with each other. For instance, H_2_ is involved in CO-induced AR development, but the concrete pathway is still unclear. Therefore, the combined effects of these compounds can mediate the induction of AR development. Besides, some target genes responsible for adventitious rooting, including *CsDNAJ-1* and *CsCDPK1/5*, could also be regulated by these small molecules.

Above all, these small-molecule compounds are involved in AR development through modifying antioxidant defenses, water balance, photosynthesis, and auxin transportation. In addition, the small compound molecules are widely and easily applied in various plant species and regulate many processes in plants, and they are more efficient and cheaper than some chemicals. Therefore, it is very meaningful to study the influence of small molecules on AR development and formation. However, the mechanism of the promotion of AR development by these small molecules has not been clear. Thus, further research is required to investigate their mechanisms. Finally, the question of whether there any other signaling compounds that can be used to promote AR formation and development should be considered. In the future, related work should be done to improve our knowledge in AR promotion and the future application of signaling molecules to horticultural products.

## Figures and Tables

**Table 1:** Overview of small-molecule compounds that induce adventitious root (AR) formation and development under no stress in plants.

Small-Molecule Compounds	Plant Species	Small Signal Molecule Mediated Effects	References
NO	Marigold	NO can trigger AR development and enhances endogenous H_2_O_2_ levels	[23]
		IAAO, POD, and PPO↑	[27]
		NR and NOS can contribute to NO production to induce AR development	[16]
	Cucumber	NO induces AR formation through NOS and NR pathways	[24,25]
		NO can trigger AR development in a cGMP-dependent manner	[17]
		NO can induce AR formation and up-regulate cell cycle-related genes	[7]
		PPO↑; POD and IAAO↓	[30]
		Two NO-releasing compounds, NOS-like and DAO, trigger AR formation	[19]
		NR and NOS promote AR development and up-regulate their gene relative expression levels	[10]
	Ground-cover chrysanthemum	PPO, IAAO, WSC, and total nitrogen↑; total polyphenol content↓	[28]
	Mountain ginseng	CAT, POD, APX, DHAR, GR, NADPH, and O_2_^−^↑	[29]
H_2_	Cucumber	H_2_ upregulates cell cycle-related genes and promotes AR formation	[7]
		50% HRW significantly induces adventitious rooting and POD, PPO, and IAAO↑	[30]
	Marigold	RWC, WSC, starch, soluble protein content, POD, PPO, and IAAO↑; stomatal aperture and electrolyte leakage↓	[5]
H_2_S	Cucumber	10 μM NaHS triggers AR development	[16]
		H_2_S can induce AR primordia	[40]
	Willow	Endogenous H2S, IAA, and NO↑	[38]
	Soybean	Endogenous H_2_S, IAA, and NO↑	[38]
CO	Mung bean	NO fluorescence↑	[41]
	Cucumber	10 μM hemin and hematin can significantly induce AR development in cucumber	[17,42]
CH_4_	Cucumber	80% MRW increases root length and number	[19]
		CH_4_-induced adventitious rooting of cucumber explants requires γ-glutamyl cysteine SGH	[40,47]
ETH	Cucumber	Exposure of cucumber explants to ETH up-regulated NOS and NR activity and their gene relative expression levels	[10]
	Marigold	IAAO, POD, and PPO↑	[27]
H_2_O_2_	Cucumber	10–50 and 20–40 μM H_2_O_2_, respectively, increases the weight and number of AR respectively	[59]
	Marigold	200 μM H_2_O_2_ significantly induces root length and root number	[23]
		IBA and H_2_O_2_ may act synergistically to mediate adventitious rooting	[26]
	Ground-cover chrysanthemum	PPO, IAAO, WSC, and total nitrogen↑; total polyphenol content↓	[28]

**Table 2:** Overview of small-molecule compounds that induce AR formation and development under stresses in plants.

Small-Molecule Compounds	Plant Species	Stress Condition	Small Signal Molecule Mediated Effects	References
NO	Cucumber	Osmotic stress	Fv/Fm, ɸPSII, qP, NPQ, SOD, CAT, and APX↑; H_2_O_2_ and O_2_^−^↓	[22]
	Marigold	Drought stress	chl (a+b) content, Fv/Fm, ɸPS II and qP, and soluble carbohydrate and protein content↑; starch content↓	[16]
	Rice	As^V^ stress	APX↑	[31]
H_2_	Cucumber	Drought stress	RWC, leaf chlorophyll content, Fv/Fm, ɸPSII and qP, SOD, POD, CAT, and APX↑	[18]
		Cd stress	DHA, GSSG, APX, DHAR, MDHAR, GR, POD, and PPO↑; MDA, H_2_O_2_, O_2_^−^, TBARS, AsA, GSH, REC, LOX, and IAAO↓	[34]
CO	Cucumber	Drought stress	leaf chlorophyll content, SOD, POD, CAT, and APX↑; RWC↓	[18]
H_2_O_2_	Marigold	Drought stress	chl (a + b) content, Fv/Fm, ɸPS II and qP, and soluble carbohydrate and protein content↑; starch content↓	[16]

**Table 3:** Overview of gene regulation by small-molecule compounds during AR formation and development.

Gene Functions	Plant Species	Small Signal Molecules-Mediated Genes	Small Signal Molecules	References
Cell cycle regulation	Cucumber	*CsCDPK1*, *CsCDPK5*	NO and ETH	[64]
		*CycA*, *CycB*, *CDKA,* and *CDKB*,	NO and H_2_	[7]
		*CsCDC6*, *CsCDPK1*, *CsCDPK5*, *and CsDNAJ-1*	CH_4_	[40,47]
Adventitious rooting-related	Cucumber	*CsDNAJ-1* and *CsCDPK1/5*	NO and ETH	[10]
		*CsDNAJ-1* and *CsCDPK1/5*	H_2_ and CO	[64]
		*CsDNAJ-1* and *CsCDPK1/5*	H_2_S and CO	[39]
		*CsDNAJ-1* and *CsCDPK1/5*	CH_4_ and CO	[65]
		*CsHO1*	NO, H_2_S, and CO	[17,62]
		*CsmiR160* and *CsmiR167*	CH_4_	[47]
Auxin-response	*Arabidopsis*	*ARF6*, *ARF 8,* and *ARF17*	ETH	[67]
	Cucumber	*CsAUX22B-*like and *CsAUX22D-*like	H_2_	[64]
		*CsAux22D-*like and *CsAux22B-*like	CH_4_	[40,47]

## Abbreviations

AR: adventitious root; NO, nitric oxide; H_2_, hydrogen gas; H_2_S, hydrogen sulfide; CO, carbon monoxide; CH_4_, methane; ETH, ethylene; H_2_O_2_, hydrogen peroxide; NR, nitrate reductase; NOS, NO synthase; SNP, sodium nitroprusside; cPTIO, 2-(4-carboxy-2-phenyl)-4, 4, 5, 5-tetramethylimidazoline-1-oxyl-3-oxide; l-NAME, *N*-nitro-l-argininemethyl ester; cGMP, cyclic guanosine monophosphate; POD, peroxidase; PPO, polyphenol oxidase; IAAO, indoleacetic acid oxidase; NADPH oxidase, triphosphopyridine nucleotide; SOD, superoxide dismutase; CAT, catalase; APX, ascorbate peroxidase; DHAR, dehydroascorbate reductase; GR, glutathione reductase; WSC, water-soluble carbohydrate; As, arsenate; HRW, hydrogen-rich water; RWC, relative water content; Cd, cadmium; MDA, malondialdehyde; O_2_^−^, superoxide radical; TBARS, thiobarbituric acid reactive substances; AsA, ascorbic acid; GSH, reduced glutathione; REC, relative electrical conductivity; LOX, lipoxygenase; DHA, docosahexaenoic acid; GSSG, oxidized glutathione; MDHAR, monodehydroascorbate reductase; NaHS, sodium hydrosulfide; HO, heme oxygenase; HO-1, heme oxygenase-1; HO-2, heme oxygenase-2; HO-3, heme oxygenase-3; PEG, polyethylene glycol; ROS, reactive oxygen species; MRW, methane-rich water; SGH, γ-glutamyl cysteine synthetase; ACC, 1-aminocyclopropane-1-carboxylic acid; IBA, indole-3-butyric acid; *CsHO1*, heme oxygenase-1; ZnPPIX, zinc protoporphyrin IX; NaN_3_, sodium azide; DAO, diamine oxidases; and HT, hypotaurine.
